# Supplementary material for: High Hydrostatic Pressure in the Modulation of Enzymatic and Organocatalysis and Life under Pressure: A Review
Source: Molecules. 2023 May 18;28(10):4172. doi: 10.3390/molecules28104172 (PMC10223222; doi:10.3390/molecules28104172)
Supplement: Supplementary file 1 [file molecules-28-04172-s001.zip › molecules-2291359-supplementary.pdf]

Table S1 Effect of high pressure on the activity of particular enzymes under pressure (MPa) and temperature (°C).

Enzyme	Source	Substrate	Pressure (MPa)	Temperature (℃)	Optimal condition	Activation volume (ml.mol^{-1})	Effect	Reference
Alcohol dehydrogenase *EC 1.1.1.1*	*Thermoanaerobium brockii*	2-pentanol	0.1-200	40	100 MPa 40 °C	-24	Activity increase up to 100 MP and decrease for pressure above 100 MPa.	[23]
		Cyclopentanol				-36		
	Thermoanaerobacter ethanolicus	2-butanol	0.1-137.5	20-52	*N*	*N*	Increasing pressure favors the formation of the S enantiomer whereas increasing temperature favors the formation of the R enantiomer	[42]
		2-pentanol				*N*		
		2-hexanol				+2.0		
Formate dehydrogenase *EC 1.2.2.1*	*Candida boidinii*	Formate	0.1 - 200	25 – 45	*N*	− 9.3	Reaction rate increases with high pressure (0.1 – 200 MPa) by one order of magnitude in neat buffer solution. The addition of TMAO and dextran further doubled this effect.	[43]
Octopine dehydrogenase *EC 1.5.1.11*	Commercial (Sigma)	Octopine	0.1-100	20	60 MPa	-16.4	The maximum activity of for octopine synthesis is at 60 MPa.	[21]
Pectin methylesterase *EC 3.1.1.11*	*Aspergillus aculeatus*	Apple pectin	100-500	20-70	200, 300 MPa 50,55 ℃	*N*	Increasing activity with elevated pressure and temperature.	[46]
	Green pepper		400-800	25,60	400-500 MPa 25 ℃	*N*	Maximum activity at 400 and 500 MPa and at 25 ℃.	[47]
	Carrot		0.1-500	20-65	500 MPa 50 ℃	-6.98	Above 40 ℃, increase in temperature clearly resulted in a gradual increase in Va; activity increased at elevated pressure and temperature up to 50 ℃ at 500 MPa.	[48]
β-glucanase *EC 3.2.1.2*	Barley malt	β-glucan	0.1-900	30-75	215 MPa 55 ℃ pH 5.6	*N*	1.6-fold higher depolymerysation of β-glucan after 20 min as compared to the max. at 0.1 MPa (45 ℃).	[49]
Cellulase *EC 3.2.1.4*	*Aspergillus niger*	Microcrystaline celulose (Avicel)	200-400	37	300 MPa 37 ℃	*N*	Activity at 300 MPa is 1.5-fold higher that at 0.1 MPa.	[55]
	CM-cellulase	Carboxymethylcellulose (CM-cellulose)	200-400	37	400 MPa 37 ℃	*N*	Activity at 400 MPa increase 1.7-fold more than at 0.1 MPa.	
	Aspergillus niger	Carboxymethylcellulose	0.1-700	30	400 MPa 30 °C	-6.33 ml mol-1 (200-400 MPa); 1.58 ml mol^{-1} (200-600 MPa) in 10%[bmim]Cl	Activity varies from 60-100% at 200-400 MPa. In 10%[bmim]Cl activity of cellulase is 1.7-fold higher (at 100 MPa) then at 0.1 MPa.	[38]

Naringinase<br>*EC 3.2.1.40*	Commercial (Sigma Aldrich)	*p*-nitrophenyl α-L-rhamnopyranoside (4-NRham)<br>*p*-nitrophenyl β-D-glucopyranoside (4-NGluc)	0.1-200	25-80	150-200 MPa<br>70-80 ℃	-7.7 (α-L-rhamnopyranoside)<br>-20 ±5.2 (naringinase)	3-fold and 4-fold increase in naringinase thermostability at 150 MPa and 70-80 ℃, respectively, compared to 0.1 MPa;<br>15-fold increase of kcat/Km values from 0.1MPa and 30 ℃ to 150 MPa and 70 ℃.	[36]
		Naringin	0.1-160	30	160 MPa<br>30 ℃	-15	Hydrolysis of naringin is accelerated at 80 MPa (5% increase) but at 160 MPa achieve maximum of activity (a 20% increase) in comparison at 0.1 MPa.	[58]
			0.1-200	25-60	160 MPa<br>30–40 ℃	-9	2-fold increase with maximum activity at 160 MPa and at 35–40 ℃.	[59]
			80-216	13-61	158 MPa<br>41 ℃<br>pH 4.0	*N*	Higher naringinase activity obtained at higher pressure and temperature with maximum activity at 41 ℃ and 158 MPa.	[60]
α-Chymotrypsin<br>*EC 3.4.21.1*	Bovine pancreas	N-succinyl-L-phenylalanine-*p*-nitroanilide	0.1-500	20-50	470 MPa<br>20 ℃	*-10*	6.5-fold higher rate up to 470 MPa at 20℃ than at 20℃ under 0.1 MPa	[7]
					360 MPa<br>50℃	-25	30-fold higher rate at 50 ℃ and 360 MPa than at 20 ℃ under 0.1 MPa.	
	Commercial (Sigma-Aldrich)	N-succinyl-L-phenylalanine-*p*-nitroanilide	0.1 - 200	20	*N*	-15	Increase in activity with the increase in pressure until 200 MPa in pure buffer and for most co-solvents.	[62]
	Commercial, immobilized on silica (Sigma-Aldrich)	Ala-Ala-Phe-7-amido-4-methylcoumarin	0.1 - 200	20	*N*	0 to -14	Product formation was increased at all pressures (up until an 8-fold increase), when compared to the reactions done under pressure with free enzyme.	[63]
Trypsin<br>*EC 3.4.21.4*	Commercial<br>(Boehringer Mannheim)	α-*N*-benzoyl-L-arginino-*p*-nitroanilid<br>(L-BAPA)	0.1-100	20	43 MPa	- 8.8	Positive effect of pressure on reaction rate up to 43 MPa.	[21]
	Commercial	BzArgOEt<br>(N-benzoyl-l- arginine ethyl ester)	0.1-100	25	100 MPa<br>25 ℃	-2.4	Activity increases up to 100 MPa.	[65]
	Commercial (Sigma)	β-lactoglobulin A (β-Ig A)	0-1 - 400	37	*N*	*N*	Pressurization up to 400 MPa, before or during enzyme action, enhanced tryptic hydrolysis of β-Ig A.	[64]
Thermolysin<br>*EC 3.4.24.27*	*Bacillus thermoproteolyticus*	3-(2-furylacryloyl)-glycyl-L-leucine amide	0.1-100	25	100 Mpa<br>25 ℃<br>6 -7 pH	-58	Activity results in over 13-fold acceleration at 100 MPa.	[22]

		(Dipeptide substrate FA-Gly-Leu-NH2)	0.1-400	20-80	250 MPa<br>40 ℃<br>7.2 pH	-44 (at 20 ℃ up to 100 MPa)<br>-48 (at 40 ℃)<br>-52.4 (at 60 ℃)	At 20 ℃ up to 100 MPa a significant 15-fold increase of reaction rate; simultaneous increase of temperature and pressure leads to acceleration of reaction up to 40-fold.	[22]
	Daiwa Kasei/Sigma	Dipeptide substrate Fua-Gly-NeuNH2	0.1-300	7-47	220 MPa	-53, -71, -86, -95 ml mol -1 at 10, 25, 35 and 45 ℃ respectively (0.1-100 MPa)	Maximum rate at around 220 MPa; at 300 MPa reaction rate is 30-fold more then at 0.1 MPa.	[67]
		Heptapeptide substrate MeOcAc-peptide			120 MPa	*N*	Maximum rate at around 120 MPa with 6-fold higher activity than at 0.1 MPa. At 300 MPa, 2-fold increase comparing with 0.1 MPa.	

N- no information
